# Soybean Toxin (SBTX) Impairs Fungal Growth by Interfering with Molecular Transport, Carbohydrate/Amino Acid Metabolism and Drug/Stress Responses

**DOI:** 10.1371/journal.pone.0070425

**Published:** 2013-07-22

**Authors:** Janne K. S. Morais, Oliver Bader, Michael Weig, Jose Tadeu A. Oliveira, Mariana R. Arantes, Valdirene M. Gomes, Maura Da Cunha, Hermogenes D. Oliveira, Daniele O. B. Sousa, Andre L. Lourencao, Ilka M. Vasconcelos

**Affiliations:** 1 Departament of Biochemistry and Molecular Biology, Federal University of Ceará, Fortaleza, Ceará, Brazil; 2 Institute for Medical Microbiology and German National Reference Center for Systemic Mycoses, University Medical Center Göttingen, Göttingen, Niedersachsen, Germany; 3 Bioscience and Biotecnology Center, State University of North Fluminense, Campos dos Goytacazes, Rio de Janeiro, Brazil; 4 Agronomic Institute of Campinas, Campinas, São Paulo, Brazil; Institute of Microbiology, Switzerland

## Abstract

Soybean toxin (SBTX) is an antifungal protein from soybeans with broad inhibitory activity against the growth and filamentation of many fungi, including human and plant pathogenic species such as *Candida albicans*, *Candida parapsilosis*, *Aspergillus niger*, *Penicillium herquei*, *Cercospora sojina* and *Cercospora kikuchii*. Understanding the mechanism by which SBTX acts on fungi and yeasts may contribute to the design of novel antifungal drugs and/or the development of transgenic plants resistant to pathogens. To this end, the polymorphic yeast *C. albicans* was chosen as a model organism and changes in the gene expression profile of strain SC5314 upon exposure to SBTX were examined. Genes that were differentially regulated in the presence of SBTX were involved in glucose transport and starvation-associated stress responses as well as in the control of both the induction and repression of *C. albicans* hyphal formation. Transmission electron microscopy showed that *C. albicans* cells exposed to SBTX displayed severe signs of starvation and were heavily granulated. Our data were indicative of *C. albicans* cell starvation despite sufficient nutrient availability in the medium; therefore, it can be speculated that SBTX blocks nutrient uptake systems. Because neither the starvation signal nor the alkaline response pathway lead to the induction of hyphae, we hypothesise that conflicting signals are transmitted to the complex regulatory network controlling morphogenesis, eventually preventing the filamentation signal from reaching a significant threshold.

## Introduction

SBTX is a 44 kDa toxic protein isolated from soybean seeds that is lethal to mice (LD_50_ = 5.6 mg^.^kg^-1^ by intraperitoneal injection). We have previously shown that SBTX inhibits the mycelial growth of several phytopathogenic species, including *Cercospora sojina* and *C. kikuchii* [1]. Both of these fungi cause economically important diseases in soybeans [2], [3]. SBTX is also active against the clinically important yeasts *Candida albicans* and *C. parapsilosis* as well as the mould *Aspergillus niger* [4]. Moreover, SBTX reduces the growth rates of *Pichia membranifaciens* and *Kluyveromyces marxianus*. In addition to inhibiting the life cycles of fungi, SBTX causes cell wall disruption, condensation and shrinkage of the cytosolic compartment, formation of pseudohyphae and cell death [4]. It is noteworthy that the concentrations of SBTX required for inhibiting the growth of phytopathogenic fungi and pathogenic yeasts are far below the doses that are lethal to mice.

To gain better insight into the mechanisms by which plant proteins act on fungi and yeasts, *C. albicans* has been used as a model system [5], [6] because many similarities exist between the cell walls of the filamentous fungi and those of yeasts [7]. *C. albicans* is a polymorphic yeast considered to be the most prevalent opportunistic fungal pathogen in humans; it causes both superficial and life-threatening deep organ infections [8]. The majority of manifestations of candidiasis at both mucosal and systemic sites are associated with the formation of biofilms on inert or biological surfaces [9], [10]. The cells growing in these biofilms can be recalcitrant to current antimicrobial treatment regimens [11]. *C. albicans* biofilm development is dependent on the formation of mycelia [12]. Mycelial cells are also a prerequisite for invasive fungal growth in tissues and therefore for infection [13]. Thus, substances that hamper the development of yeast biofilms and/or the formation of mycelial cells are valuable as potential new drugs for the treatment microbial diseases [14].

Elucidating the mechanism by which SBTX acts on fungi and yeasts may therefore contribute to the design of novel antifungal drugs and/or to the development of transgenic plants resistant to pathogens. Therefore, the present study was undertaken to shed light on the mechanism of action of SBTX by using an oligonucleotide DNA microarray approach to identify differentially expressed genes. Additionally, transmission electron microscopy was employed to visualise ultrastructural alterations associated with the antifungal effects of SBTX on cells from *C. albicans* strain SC5314.

## Materials and Methods

### *C. albicans* growth conditions

*C. albicans* SC5314 was used in this study as a reference strain. Yeast cells were grown in Sabouraud's dextrose broth (SDB). Like many antifungal proteins and peptides, SBTX is ineffective in full-strength medium [15]. Therefore, for experiments involving treatment with the protein, a four-fold diluted SDB was used, allowing the protein to be fully active without significantly affecting the growth of any of the *C. albicans* strains used for this study. SBTX was purified as described previously [1]. Briefly, soybean defatted powder was extracted with 0.025 M Tris-HCl/0.005 M dithiothreitol (DTT), pH 7.5, (1∶5, m/v) for 3 h at 4°C under constant stirring and filtered through cheesecloth. The press cake was re-extracted for 2 h under the same conditions. After centrifugation at 21,000 *g* for 30 min at 4°C, the supernatant, denoted as the crude extract, was fractionated by saturation to 20-55% with solid ammonium sulphate. The precipitated proteins were dissolved in and dialysed against the extracting buffer and applied to a DEAE-cellulose column equilibrated with the same buffer. After elution of the unbound proteins, they were concentrated by precipitation with 90% ammonium sulphate, exhaustively dialysed against 0.025 M Tris-HCl/0.005 M DTT, pH 7.5, and applied to a CM-Sepharose column equilibrated with the above-mentioned buffer. The SBTX enriched fraction was eluted with 0.2 M NaCl added to the equilibrating buffer, concentrated with 90% ammonium sulphate, dialysed against 0.025 M Tris-HCl, pH 7.5, and applied to a Superdex 200 HR 10/30 column equilibrated with 0.025 M Tris-HCl containing 0.5 M NaCl, pH 7.5, from which the purified SBTX was obtained.

### Treatment of *C. albicans* SC5314 with SBTX

Yeast growth inhibition was performed following a protocol modified from Broekaert et al. [16]. The cells (1×10^4^ cells per 1.0 mL of 0.15 M NaCl) were incubated at 30°C in 200 µL microplate wells in the presence or absence of SBTX at final concentrations ranging from 50–400 µg•mL^−1^ in SDB/4. Optical readings at 600 nm were taken at time zero and every 15 min for 24 h. Each pair of samples (untreated and SBTX-treated cells) constituted a single experiment and three biologically independent experiments were performed.

For transcriptional profiling, the cells were grown in SDB/4 in the presence or absence of SBTX (200 µg•mL^−1^). The cultures were grown until they reached an OD_600_ of approximately 0.5, after 16 and 18 h, for untreated and SBTX-treated cells. The cells were harvested by centrifugation (3000 *g* at 25°C), snap-frozen in liquid nitrogen and stored at −80°C. Each pair of samples (untreated and SBTX-treated cells) constituted a single experiment and two biologically independent experiments were performed.

### Extraction of total RNA

RNA was extracted with an RNeasy Mini Kit (Qiagen, Düsseldorf, Germany) according to the manufacturer's instructions. The samples were quantified using standard spectrophotometry; an A260/280 ratio>1.8 was considered acceptable. The quality of the total RNA was determined with an Agilent 2100 Bioanalyser (Agilent Technologies, Palo Alto, CA). The samples were utilised for further studies if the ribosomal 28S and 18S RNA bands were present.

### Microarray hybridisation

Microarrays were performed using the Low RNA Input Linear Amplification Kit Plus One-Color Protocol (Agilent Technologies, Waldbronn, Germany) and an Agilent One-Color RNA Spike-in Kit (Agilent Technologies, Waldbronn, Germany) following the manufacturer's standard procedure. Global gene expression analysis was performed using the *Candida* project custom 4×44 K design array (Agilent) [17] to span the complete open reading frame (ORF) set from assembly 21 (www.candidagenome.org) of the *C. albicans* genome (NCBI accession number GSE47364). A 600 ng sample of total RNA was used as starting material to prepare cDNA. cDNA synthesis and *in vitro* transcription (IVT) were performed according to the manufacturer's recommendations. The quantity and quality of the labelled amplified cRNA were determined using the NanoDrop ND-1000 UV-VIS Spectrophotometer (version 3.2.1). The hybridisations were performed for 17 h at 10 rpm and 65°C in a hybridisation oven (G2545A; Agilent Technologies, Waldbronn, Germany). Washing and staining of the arrays were performed according to the manufacturer's instructions. Cy3 intensities were detected by one-color scanning using an Agilent DNA Microarray Scanner (G2505B; Agilent Technologies, Waldbronn, Germany) at 5-micron resolution. The scanned image files were visually inspected for artefacts and subsequently analysed.

### Microarray data analysis

Intensity data were extracted using Agilent's Feature Extraction (FE) software (version 9.5.3.1). A quality control based on internal controls using Agilent's GE1_107_Sep09 protocol was included. All chips passed the quality control and were analysed using the Limma package [18] of Bioconductor [19]. The data reported here were generated in compliance with the MIAME guidelines and have been deposited in the Candida Genome Database, which is freely accessible at www.candidagenome.org. A reproducible fold-change of≥1.5 was used as a criterion for upregulation or downregulation [20]. Pathway analysis was performed with the tools available from candidagenome.org.

### Quantitative real-time RT-PCR assay

Total RNA (1 µg) was reverse-transcribed in a 25 µL reaction volume into first-strand cDNA using Superscript III and random hexamers (Invitrogen, Darmstadt, Germany). An aliquot (3 µL) of cDNA mix was added to 12.5 µL of iQ™SYBR® Green Supermix (Bio-Rad Laboratories, Hercules, CA) and 375 ng of primer solution in a final volume of 25 µL per reaction. The amplification efficiency was assessed using LinRegPCR [21]. The corresponding primer sequences are listed in Table 1. Real-time PCR analysis was performed in a Bio-Rad iCycler iQ5 (Bio-Rad, Munich, Germany) using the following cycling parameters: 10 min at 95°C, 40 cycles of 15 sec at 95°C and 1 min at 60°C. The resulting Ct values were normalised based on the mean of 18S rRNA gene expression.

**Table 1:** List of primers used for quantitative RT-PCR.

Target genes	Primer pairs (5′ to 3′)	Amplicon size (bp)
*RIM101*	fwd: 5′- AAGCGAGCAAACTCGATGAA -3′	118
	rev: 5′- GTGGAGCTTGTGCCATTTGT -3′	
*AOX2*	fwd: 5′- TGTTGGTCAAGGGGTTTTCA -3′	160
	rev: 5′- TGCCACTTCAGGGATTTTCA -3′	
*TUP1*	fwd: 5′- GTACAACCCAGCGTTCTCCA -3′	140
	rev: 5′- CAACTCTCCGGTGGTGACAT -3′	
*HGT1*	fwd: 5′- GGGCCACATTAATGGTGTTG -3′	145
	rev: 5′- CGATCACCATGAGCTTGGAT -3′	
18S rRNA	fwd: 5′- GACACGGGGAGGTAGTGACA -3′	120
	rev: 5′- GGGTCCAGTACGCATCAAAA -3′	

### Assessment of SBTX activity on the growth of *C. albicans* mutants

To assess whether deletion of the *RIM101* or *TUP1* genes enhanced or reduced the sensitivity of these yeast strains to SBTX, *C. albicans* mutant *tup1Δ/tup1Δ* [22] and *C. albicans* mutant *rim101Δ/rim101Δ* [23] cells were incubated with SBTX (200 µg·mL^−1^ final concentration) following the protocol described above. After incubation (36 h), the cells were evaluated under an optical microscope (Zeiss Axiovert 135, Carl Zeiss AG, Switzerland). Micrographs were captured with a digital camera (Sony, MCV-CD350 model, 3.2 megapixels).

### Transmission electron microscopy (TEM)

Wild type yeast cells were grown for 48 h in Sabouraud broth in the presence (400 µg·mL^−1^) or absence of SBTX. After incubation, the *C. albicans* cells were harvested and fixed in 2.5% (v/v) glutaraldehyde and 4% (v/v) paraformaldehyde in 0.05 M cacodylate buffer, pH 7.2, for 30 min at 25°C. After fixation, the samples were washed and post-fixed in 1% (m/v) osmium tetroxide in the above buffer for 1 h at 25°C. The samples were dehydrated in a graded acetone series (30, 50, 70, 90 and 100%, v/v) and embedded in Epon resin (Polybeded). Ultrathin sections (0.1 µm thickness) were cut using an ultramicrotome (Ultracut E 70 17 04, Reichert-Jung, Austria), fixed onto copper grids and stained with uranyl acetate for 10 min followed by lead citrate for 5 min. Visualisation of the cells was performed with a transmission electron microscope (ZEISS TEM 900) at 80 kV.

## Results

### Evaluation of SBTX-mediated *C. albicans* growth inhibition

**Figure 1:**
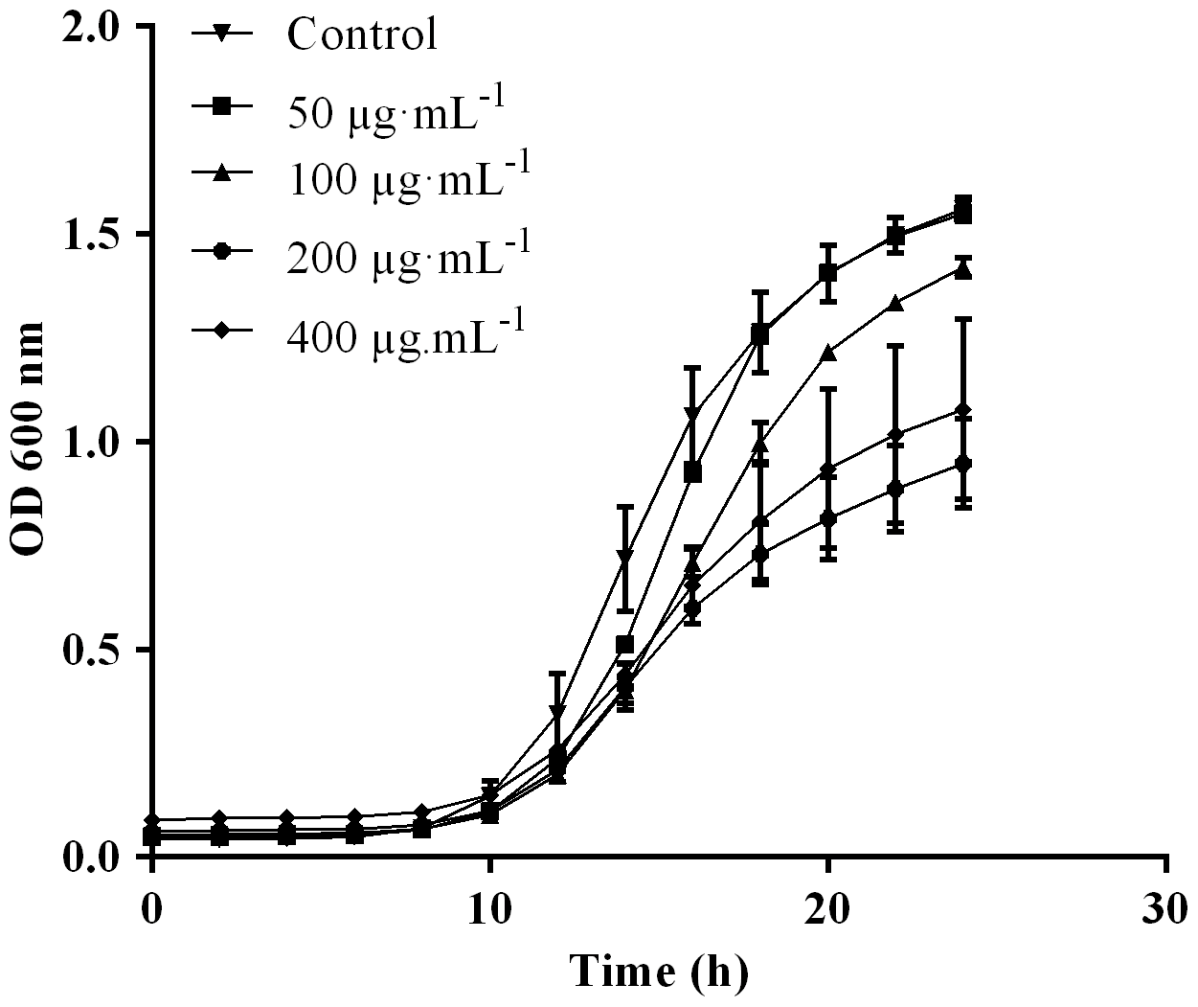
Growth inhibition assay of *C. albicans* exposed to SBTX (50–400 µg·mL^−1^). Fungal growth was measured based on absorbance at 600 nm, as described in the Materials and Methods. The control corresponds to cells grown in the absence of SBTX. Each point represents the mean of three estimates. The standard deviation was less than 10%.

SBTX prepared from soybeans inhibited the growth of *C. albicans* at concentrations ranging from 50-400 µg•mL^−1^ (Figure 1). The SBTX batch prepared during this study inhibited approximately 50% of the growth of *C. albicans* at a concentration of 200 µg•mL^−1^ (Figure 1). Therefore, this subinhibitory concentration of SBTX was used in subsequent experiments. Previous control experiments showed that other proteins (e.g., BSA) do not elicit this effect under these conditions [24].

### Changes in gene expression in response to SBTX exposure

A total of 39 genes were upregulated and 22 genes were downregulated in response to 16 h of SBTX treatment. After 18 h, 51 genes displayed altered expression; some of these genes overlapped with the genes that displayed altered expression after 16 h of SBTX treatment. Table 2 shows select genes that were upregulated in response to SBTX exposure for 16 h and 18 h. For quality control, qRT-PCR was conducted using four genes that were highly upregulated in the microarray analysis, namely *RIM101, TUP1, AOX2* and *HGT1*. The upregulation of these genes was confirmed by qRT-PCR. The expression levels of these genes were found to be increased by 123.64-fold, 25.46-fold, 13.83-fold and 8.63-fold (absolute values), respectively, after 16 h.

**Table 2:** Selected *C. albicans* genes upregulated in response to SBTX exposure.

Gene ontology (GO)/genes†	Gene name†	Fold change‡				
		16 h		18 h		
*Small molecule transport (13 out of 985 genes)*						
High-affinity glucose transporter	*HGT1*	2.28			5.31	
Putative glucose transporter	*HGT7*	3.69			-	
Putative glucose transporter	*HGT2*	2.24			4.78	
Basic amino acid permease	*CAN1*	2.53			-	
High-affinity glucose transporter	*HXT5*	-			3.73	
Putative nicotinic acid transporter	*TNA1*	2.81			-	
Protein similar to amino acid permeases	*GAP2*	-			2.47	
Putative high-affinity maltose transporter	*MAL31*	1.90			-	
Glycerol permease	*HGT10*	-			1.85	
Putative peroxisomal ubiquitin conjugating enzyme	*PEX4*	1.91			-	
Putative Golgi v-SNARE	*SFT1*	1.73			-	
Putative nicotinic acid transporter	*TNA1*	2.81			-	
Essential protein involved in endocytosis	*PAN1*	-			2.36	
*Carbohydrate metabolic process (9 out of 251 genes)*						
UDP-glucose 4-epimerase	*GAL102*	-			1.87	
Phosphoenolpyruvate carboxykinase	*PCK1*	-			1.75	
UDP-glucose 4-epimerase	*GAL10*	1.78			2.54	
Putative galactose-1-phosphate uridyl transferase	*GAL7*	-			1.92	
Galactokinase	*GAL1*	-			1.82	
Mitochondrial ADP/ATP carrier protein	*PET9*	1.90			-	
Isocitrate lyase	*ICL1*	-			3.22	
3-hydroxyacyl-CoA epimerase	*FOX2*	2.35			-	
Malate synthase	*MLS1*	1.94			-	
*Stress response (7 out of 778 genes)*						
Transcription factor involved in alkaline pH response	*RIM101*	2.30				-
Calcineurin-regulated C2H2 zinc-finger transcription factor	*CRZ1*	1.93				-
UDP-glucose 4-epimerase	*GAL10*	1.78				2.54
Transcriptional corepressor	*TUP1*	-				1.96
Adhesin	*ALS1*	-				1.69
Adhesin	*ALS4*	-				2.16
Small heat shock protein involved in stress response	*HSP21*	-				2.65
*Cellular respiration (2 out of 89 genes)*						
Alternative oxidase	*AOX2*	3.9				4.77
Mitochondrial ADP/ATP carrier protein	*PET9*	1.9				-
*Filamentous growth (7 out of 540 genes)*						
UDP-glucose 4-epimerase	*GAL10*		1.78	2.54		
Transcription factor involved in alkaline pH response	*RIM101*		2.30	-		
Cyclin-dependent protein kinase regulator activity	*PCL5*		2.04	-		
Transcriptional corepressor	*TUP1*		-	1.96		
Putative serine/threonine kinase	*SHA3*		-	1.72		
Adhesin	*ALS1*		-	1.69		
Adhesin	*ALS4*		-	2.16		
*Arginine biosynthesis (2 out of 129 genes)*						
Argininosuccinate synthase	*ARG1*		1.90	-		
Putative ornithine carbamoyltransferase	*ARG3*		1.86	-		
*Others (31 out of 459 genes)*						
16 genes of unknown function or others			>1.5			
15 genes of unknown function or others				>1.5		

† Gene names and gene ontology according to the *Candida albicans* genome database (CGD). ‡ Absolute values>1.50 indicate that genes were upregulated in *C. albicans* in the presence of SBTX compared with *C. albicans* cultured without SBTX.

**Figure 2:**
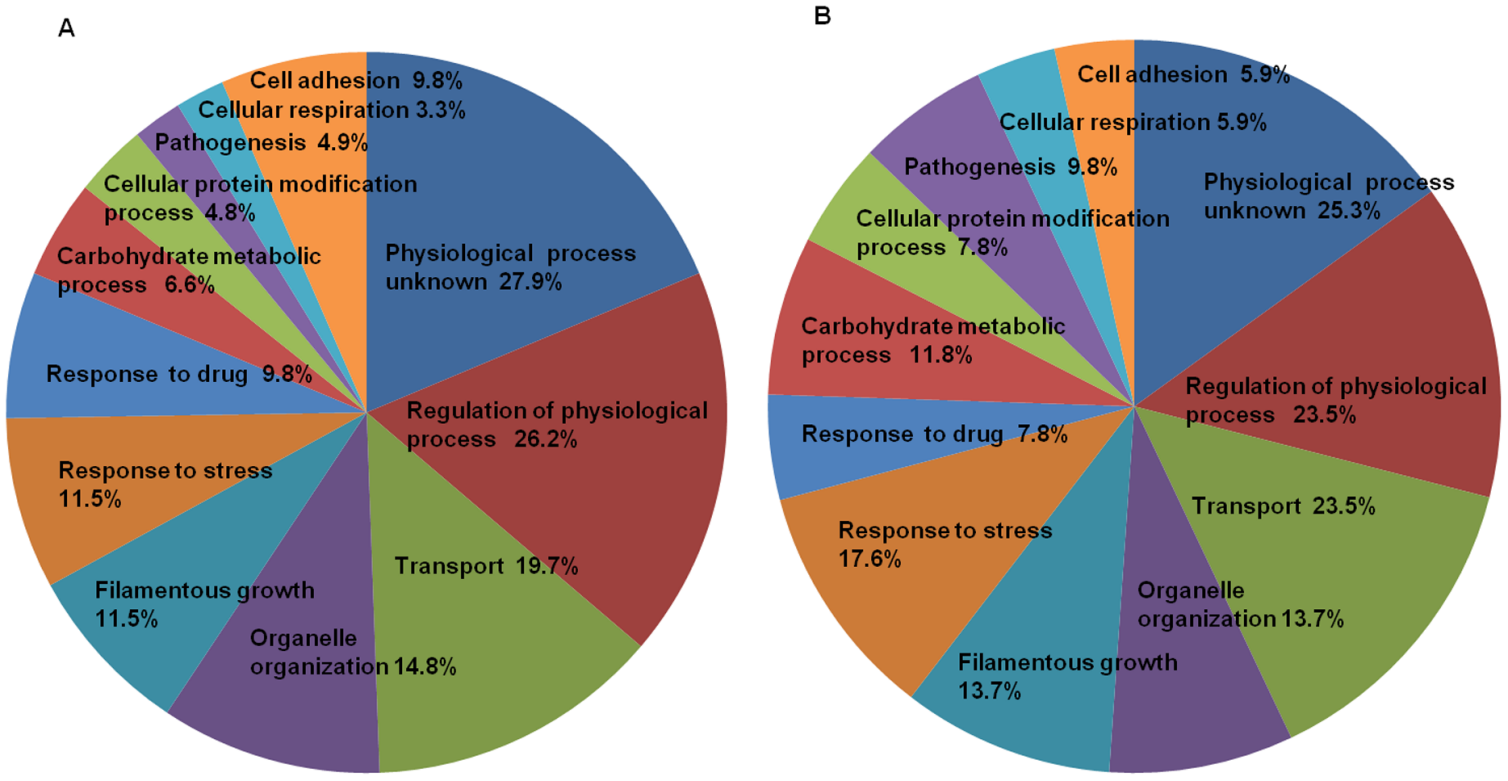
Functional categories of differentially expressed genes upon treatment with SBTX. Pie charts showing particular categories of *C. albicans* genes that were differentially expressed upon treatment with SBTX at 200 µg·mL^−1^ for 16 h (A) and 18 h (B). A total of 61 genes were clustered for 16 h and 51 genes were clustered for 18 h. The Candida Genome Database (CGD) gene ontology slim mapper was used to cluster these differentially expressed genes into categories (http://www.candidagenome.org/).

Among the 61 genes that were differentially expressed at 16 h, 26.2% (16 genes) were associated with the regulation of biological processes. Of particular interest were those involved in transport (19.7%, 12 genes), organelle organisation (14.8%, 9 genes), filamentous growth (11.5%, 7 genes), response to stress (11.5%, 7 genes), response to drugs (9.8%, 6 genes) and carbohydrate metabolism (6.6%, 4 genes) (Figure 2A). Among the upregulated genes (Table 2), 9 were involved in small molecule (mainly hexose) import. Other metabolic processes affected were gluconeogenesis (*PCK1*) and galactose utilisation (*GAL10*). Moreover, the gene expression data showed that in the presence of SBTX, genes involved in stress responses and/or filamentation (e.g., *PCL5*, *RIM101*, *CRZ1* and *GAL10*) were upregulated. Among the downregulated genes (Table 3) were those involved in the cell cycle and cell surface (e.g., *PCL2*, *PES1*), amino acid and RNA metabolic processes (e.g., *NUP49*), cellular respiration (e.g., *TAR1*) and filamentous growth (e.g., *NOP15*).

**Table 3:** Selected *C. albicans* genes downregulated in response to SBTX exposure.

Gene ontology (GO)/genes†		Gene name†	Fold change‡		
			16 h		18 h
*Cellular cycle and cell surface (6 out of 400 genes)*					
Cyclin homolog		*PCL2*	0.57		-
Pescadillo homolog		*PES1*	0.54		-
Putative histone H3		*HHT2*	-		0.37
Putative histone H3		*HHT21*	-		0.35
Putative histone H4		*HHF1*	-		0.47
Putative GPI-anchored protein		*PGA61*	-		0.52
*RNA metabolic processes (4 out of 723 genes)*					
Phosphoribosylanthranilate isomerase		*TRP1*	0.58	-	
Putative T subunit of glycine decarboxylase		*GCV1*	-	0.57	
Nuclear pore protein		*NUP49*	0.41	-	
Pescadillo homolog required for filament-to-yeast switching		*PES1*	0.54	-	
*Cellular respiration (2 out of 89 genes)*					
Component of mitochondrial ribosome		*MRP20*	0.49	-	
Ortholog of *S. cerevisiae* Tar1p		*TAR1*	0.34	-	
*Filamentous growth (3 out of 540 genes)*					
Pescadillo homolog required for filament-to-yeast switching		*NOP7*	0.54	-	
Nucleolar ribosome biogenesis factor		*NOP15*	0.37	-	
Protein required for growth in medium lacking phosphate		*PHO4*	0.61	-	
*Pathogenesis (1 out of 215 genes)*					
Cytoplasmic protein		*WH11*	-		0.50
*DNA metabolic process (3 out of 366 genes)*					
Involved in DNA replication		*PSF1*	-		0.55
Putative single-stranded DNA-binding protein		*RIM1*	-		0.49
Putative adenylate kinase		*ADK1*	0.49		-
*Ribosome biogenesis (3 out of 283 genes)*					
Nucleolar ribosome biogenesis factor		*NOP15*	0.37		-
Nuclear pore protein		*NUP49*	0.41		-
Predicted ribosomal protein		*RPL82*	0.56		-
*Organelle organisation (1 out of 918 genes)*					
Component of mitochondrial ribosome		*MRP20*	0.49		-
*Others (12 out of 459 genes)*					
7 genes of unknown function and others			<1.50		
5 genes of unknown function and others					<1.50

† Gene names and gene ontology according to the *Candida albicans* genome database (CGD). ‡ Absolute values<1.50 indicate that genes were downregulated in *C. albicans* in the presence of SBTX compared with *C. albicans* cultured without SBTX.

Of the 51 genes differentially expressed after 18 h, 23.5% (12 genes) were involved in the regulation of physiological process, 23.5% (12 genes) were involved in transport, 17.6% (9 genes) were involved in stress responses and 13.7% (7 genes) were involved in filamentous growth (Figure 2B). In addition to the genes that displayed differential regulation at 16 h, the filamentation-associated genes *TUP1*, *ALS4*, *SHA3* and *ALS1* were upregulated at 18 h (Table 3). Genes additionally downregulated were indicative of the transition of the culture to stationary phase (e.g., *PSF1, RIM1, HHT2, HHT21* and *HHF1)*.

### Assessment of SBTX activity on the growth of *C. albicans* gene deletion mutants

**Figure 3:**
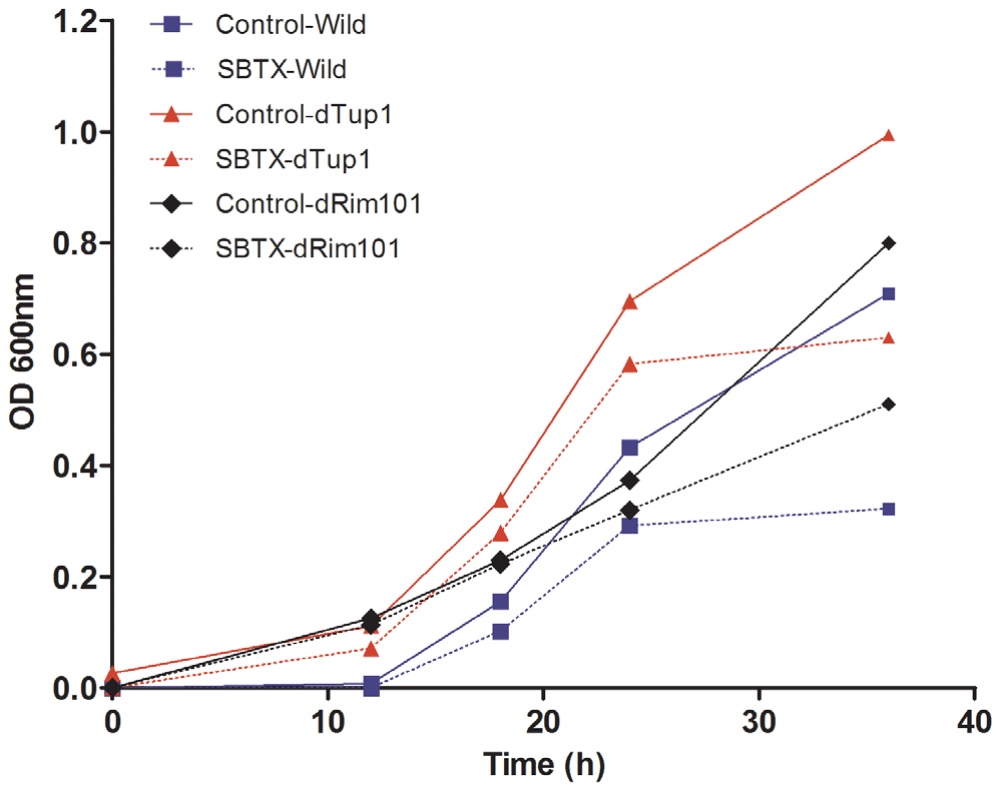
Growth curves of wild type and mutant *C. albicans* strains in the presence of SBTX. Wild type and mutant (*tup1Δ/tup1Δ* and *rim101Δ/rimΔ*) *C. albicans* strains were grown for 40 h in the presence or absence of SBTX (200 µg·mL^−1^).

**Figure 4:**
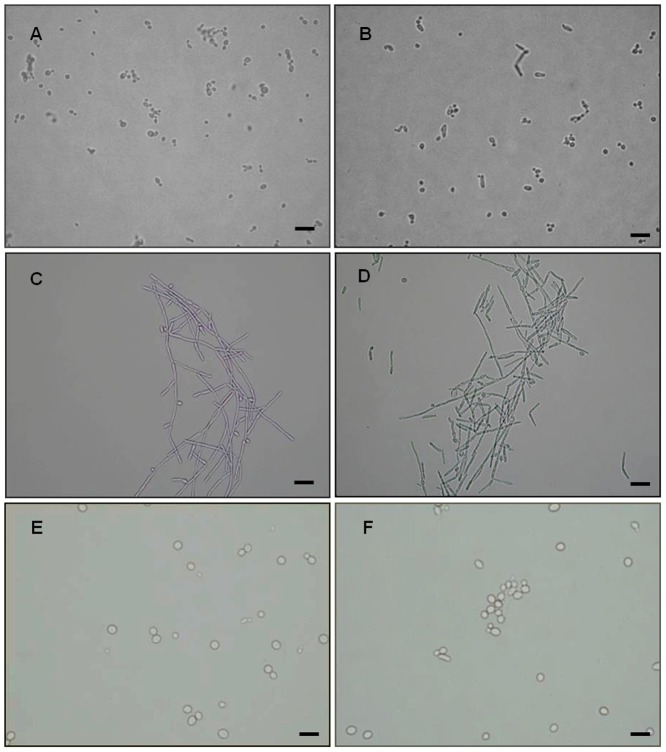
Light micrographs of wild type and mutant *C. albicans* strains in the presence of SBTX. Cells were incubated in the absence of SBTX (A, C, E) or in the presence of SBTX (B, D, F) (200 µg·mL^−1^). *C. albicans* wild type strains (A, B), the *C. albicans tup1Δ/tup1Δ* mutant (C, D) and the *C. albicans rim101Δ/rimΔ mutant* (E, F) are shown. Bars (A-F): 10 µm.

It has been well documented that SBTX inhibits the growth of *C. albicans* wild type strains [5]. SBTX-induced growth inhibition was also observed in *C. albicans tup1Δ/tup1Δ* (Figure 3B) and *rim101Δ/rim101Δ* deletion strains (Figure 3C). No SBTX-induced morphological changes were observed in wild type (Figure 4B), *tup1Δ/tup1Δ* (Figure 4D) or *rim101Δ/rim101Δ* (Figure 4F) cells when compared with their respective parental control strains (Figure 4A, C, E).

### SBTX-induced ultrastructural alterations in *C. albicans* cells

**Figure 5:**
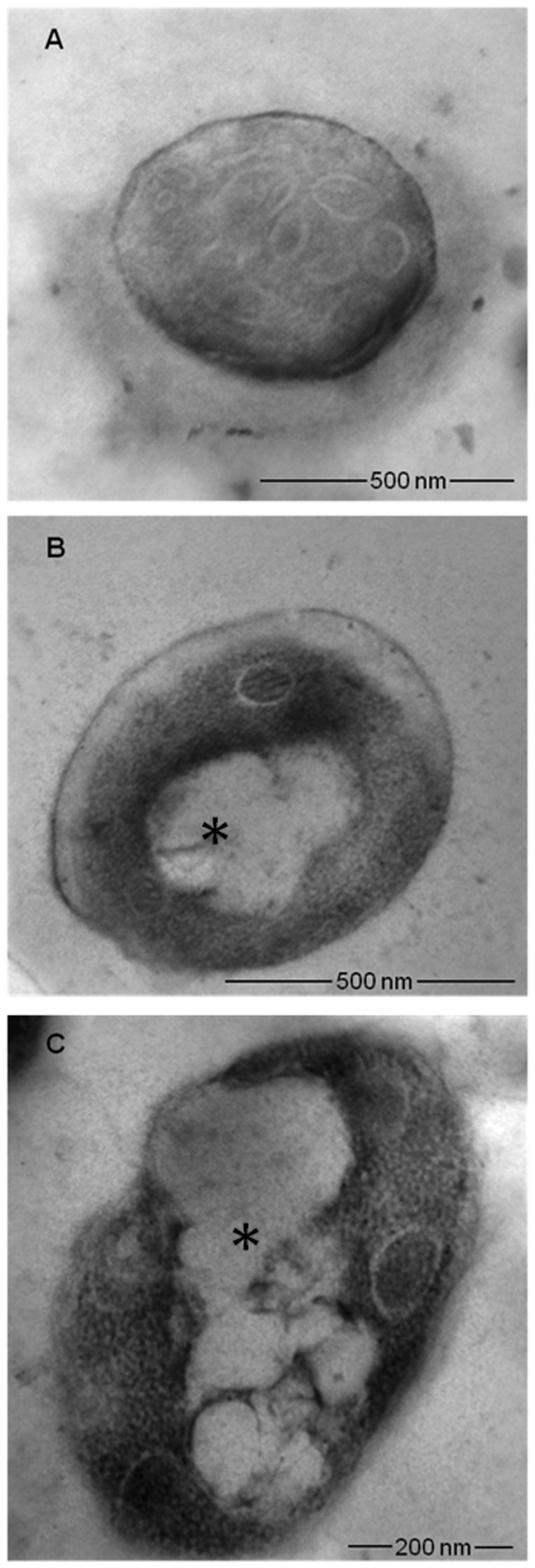
Transmission electron microscopy (TEM) of *C. albicans* in the presence of SBTX. Representative micrographs of single cells observed by TEM of *C. albicans* cultured in the absence (A) or presence (B, C) of SBTX (400 µg·mL^−1^). Asterisks indicate condensation and shrinkage of a heavily granulated cytosol and increased vacuolisation in *C. albicans* treated with SBTX.

TEM of wild type cells revealed condensation and shrinkage of a heavily granulated cytosol and increased vacuolisation in SBTX-treated (400 µg•mL^−1^) *C. albicans* cells. Structural disorganisation and loss of cytoplasmic content were also observed in SBTX-treated cells (Figure 5B, C) when compared with control cells (Figure 5A).

## Discussion

Previously, we showed that SBTX inhibited morphological development in plant and human pathogenic fungi and that the presence of SBTX increased the membrane permeability of fungal cells [5]. In this work, we used TEM analysis of *C. albicans* cells to show that prolonged exposure to SBTX resulted in condensation and shrinkage of a heavily granulated cytosol, increased vacuolisation, loss of normal cell structure and loss of cytoplasmic content. The SBTX-induced modifications in *C. albicans* were even more prominent than those observed in *P. membranifaciens* [5].

To further investigate the transcriptional basis for the effects induced by SBTX and to shed light on its mechanism of action, gene expression analysis was performed on SBTX-treated and untreated *C. albicans* SC5314. Under the conditions investigated, neither culture produced hyphae and the SBTX-treated culture reached stationary phase at an OD_600_ that was approximately 50% of that at which untreated cells reached stationary phase. At the 18 h time point, several indicators of the transition to stationary phase were observed in the SBTX-treated cells, e.g., the downregulation of *PSF1, RIM1, HHT2, HHT21* and *HHF1.* As expected from the TEM analysis and previous phenotypic results, pathway analysis of differentially expressed genes during late log phase showed that several morphogenesis-related pathways and general stress responses were differentially regulated. Furthermore, nutrient sensory and uptake pathways were differentially activated in untreated and SBTX-treated cells.

Our first observation was that several starvation signals were activated. Intracellular levels of glucose appeared to be low, as the high-affinity glucose transporter *HGT1* [25] was activated and several other Mig1-regulated genes were derepressed. This derepression was most dramatic for enzymes of the Leloir pathway (*GAL1* and *GAL10*). Additionally, genes involved in other metabolic pathways indicating starvation were differentially expressed: Maltose (*MAL31*) and glycerol import (*HGT10*) were activated, gluconeogenesis was induced as indicated by *PCK1* derepression under low intracellular glucose levels [26], [27], glyoxylate cycle genes (*ICL1* and *MLS1*) were activated and the gene encoding 3-hydroxyacyl-CoA epimerase (*FOX2*), an enzyme essential in lipid oxidation, was also induced, indicating that exposure of *C. albicans* to SBTX must have led to fatty acid metabolism. Nevertheless, genes activated by the high extracellular glucose sensor Hgt4 [28], namely *HGT7*, *HXT5* and *AOX2*, as well as the glucose transporter *HGT2* remained transcriptionally active. This finding indicated the presence of sufficient glucose in the medium, which was also in accordance with the less dense growth of the culture compared with the control.

One significant difference between the cultures was that the SBTX-containing culture had high levels of protein (SBTX itself) as an additional carbon and nitrogen source, which may have interfered with glucose sensing. Indeed, *CAN1* and *GAP2,* both of which encode basic amino acid permeases, were upregulated in the presence of SBTX. However, no other transcriptional indicator of increased protein utilisation was found and the expression of secretory protease was unaltered (data not shown). Similarly, in previous control experiments [24], other proteins did not elicit the morphological effects induced by SBTX, indicating that the effects observed here were specific to SBTX and not a general response to protein utilisation. Taken together, these results show that the metabolic genes differentially regulated in our experiments are indicative of cell starvation despite the availability of sufficient nutrients in the medium. This is in agreement with the morphological alterations observed in TEM sections.

In *C. albicans*, starvation signals such as Mig1 derepression would normally trigger filamentation [29]; however, the cells failed to do so. In our transcriptional analysis, we observed the upregulation of the three central regulatory morphogenic factors, *RIM101, CRZ1* and *TUP1,* in the presence of SBTX. Rim 101 is a transcription factor that is proteolytically activated upon neutral to alkaline pH sensory input and triggers, among other processes, filamentation in *C. albicans* [30], [31]. In *Saccharomyces cerevisiae*, the presence of SBTX inhibited culture medium acidification [5]. Together with the increased transcription of *RIM101* and *CRZ1* [32], this is suggestive of a neutral to alkaline pH sensory input in *C. albicans*. Considering that the SBTX-induced growth inhibitory effect was less pronounced in the *C. albicans rim101Δ*/*rim101Δ* mutant, the regulatory event preventing this signal most likely does not lie in the Rim101 activation cascade but, rather, occurs further downstream.

On a molecular level, the failure to produce hyphae was evident through increased transcription of the gene for the morphogenic repressor Tup1. Tup1 acts in concert with Mig1 and Nrg1, which divide morphogenesis-responsive genes into subsets [29]. None of the singly or doubly Tup1/Nrg1-dependent genes were upregulated; all genes derepressed from this regulon were Mig1-dependent. In the *C. albicans tup1Δ/tup1Δ* mutant, SBTX growth inhibitory activity was reduced, showing that Tup1 is, at least in part, necessary for the inhibitory activity of SBTX. Similarly, filamentous growth was observed in *tup1Δ/tup1Δ* cells in the presence of SBTX, confirming the role of the *TUP1* gene in the suppression of the filamentous growth of *C. albicans* under these conditions.

Additionally, many of the genes found to be differentially regulated are part of the Hap43 regulon [33]. This observation raises the possibility that iron starvation may contribute to the underlying cellular dysfunction in SBTX-treated cells.

**Figure 6:**
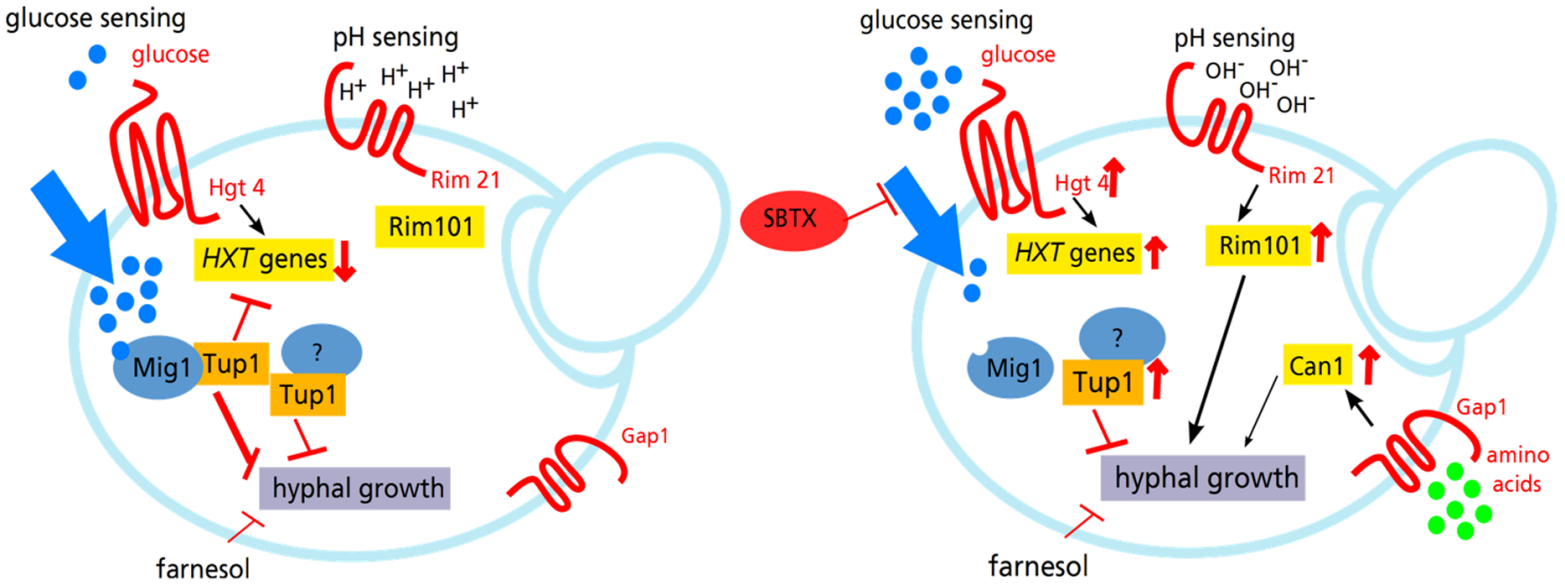
Hypothetical model for SBTX-induced signals in *C. albicans*. In the presence of SBTX (right panel), nutrient uptake is blocked, leading to cell starvation. The presence of sufficient nutrients in the medium may lead to conflicting morphogenic signals compared with untreated cells (left panel), eventually preventing hyphal growth. Blue dots: glucose; green dots: amino acids; red arrows: upregulation or downregulation; red bars: inhibition; black arrows: activation.

In summary, the results reported in the present study suggest a model for the molecular mechanism of action of SBTX in *C. albicans* (Figure 6). SBTX likely crosses the cell wall, affects the glucose sensor Hgt4, which is present in the cell membrane, and blocks nutrient uptake, causing starvation. However, although various ultrastructural alterations were observed by TEM, neither the starvation signal nor the activation of the alkaline response pathway triggered the production of hyphae. The most likely explanation for this finding is that, due to the presence of sufficient nutrients in the medium, conflicting signals are transmitted to the complex regulatory morphogenic network, eventually preventing the filamentation signal from reaching a significant threshold.
